# Staged, blood-sparing management of postmyocardial infarction ventricular septal rupture in a Jehovah's Witness

**DOI:** 10.1016/j.xjtc.2025.102173

**Published:** 2025-12-03

**Authors:** Yuichiro Fukumoto, Chiaki Aichi, Yusuke Imamura, Mototsugu Tamaki, Yasuhide Okawa, Hisao Suda, Hideki Kitamura

**Affiliations:** a Department of Cardiovascular Surgery, Nagoya Heart Center, Nagoya, Japan; b Department of Cardiovascular Surgery, Nagoya City University, Graduate School of Medical Sciences, Nagoya, Japan

**Figure:**
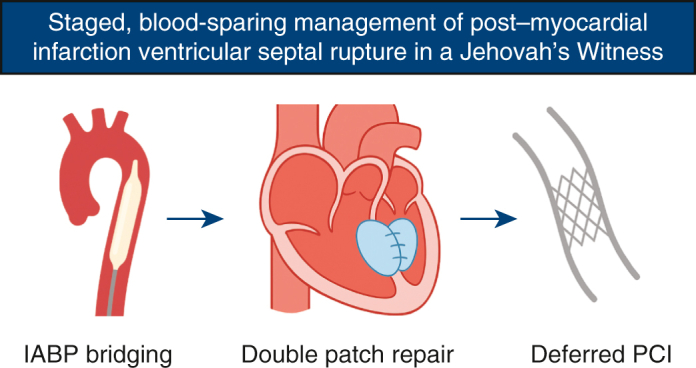
Blood-sparing VSR repair after MI in a Jehovah's Witness: IABP bridge and deferred PCI.

**Central Message**

In a Jehovah's Witness with post-MI VSR, IABP bridging, blood-sparing patch repair, and deferred PCI achieved transfusion-free recovery.

Postinfarction ventricular septal rupture (VSR) remains one of the most lethal mechanical complications of acute myocardial infarction (MI), with an incidence ≈0.2% and in-hospital mortality >40% despite reperfusion.^1^ For patients who are Jehovah's Witness (JW), refusal of transfusion complicates timing and perioperative management. We report transfusion-free repair using intra-aortic balloon pump (IABP) bridging, meticulous blood-sparing conduct, and deferred percutaneous coronary intervention (PCI).

## Clinical Summary

**Figure 1:**
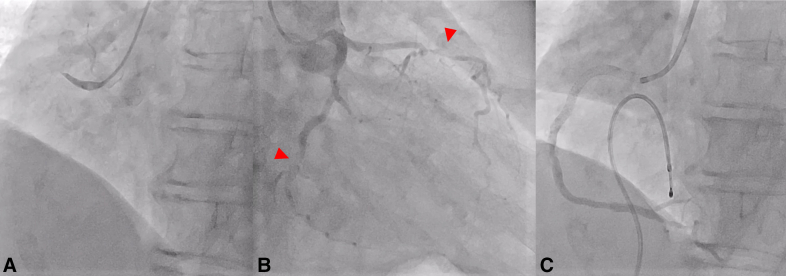
Preoperative coronary angiography shows (A and B) culprit 1 occlusion with severe residual disease in 7 (90%) and 14 (90%) (*arrowheads*) and (C) restoration of Thrombolysis In Myocardial Infarction 2 flow after primary percutaneous coronary intervention.

A 75-year-old JW woman with hypertension, dyslipidemia, and smoking history presented 4 hours after symptom onset with inferior ST-segment elevation; she was resuscitated after transient ventricular fibrillation. Coronary angiography identified #1 culprit with critical residual disease in the left coronary system (Figure 1, *A* and *B*); flow improved to TIMI 2 after primary PCI to the right coronary artery (Figure 1, *C*).

**Figure 2:**
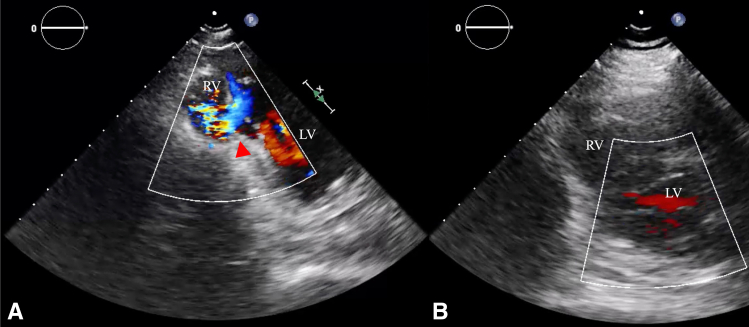
Apical short-axis transthoracic echocardiography: (A) preoperative left-to-right shunt across an apical ventricular septal defect (*arrowhead*); (B) postoperative absence of residual shunt. *RV*, Right ventricle; *LV*, left ventricle.

On day 6 after PCI, she developed back pain and a new systolic murmur. Echocardiography demonstrated a left-to-right shunt from the mid-septum to the apex (Qp/Qs = 1.8) (Figure 2, *A*), confirming post-MI VSR. Given transfusion refusal and hemoglobin 9.7 g/dL, we adopted a staged, blood-sparing plan: we optimized erythropoiesis with intravenous iron and erythropoietin, minimized phlebotomy, and proceeded to repair once hemoglobin and organ function improved, deferring residual revascularization. A pulmonary artery catheter was placed, and continuous hemodiafiltration was used for the first 4 days. She remained awake and not intubated. IABP was inserted via the right femoral artery from the onset, precluding ambulation. It was temporarily removed after stabilization to promote hematologic recovery and limit deconditioning but reinserted on day 10 for hemodynamic collapse requiring vasopressors. After a second removal, recurrent circulatory failure occurred, leaving no further opportunity for delay; surgery was performed on day 23 with hemoglobin 11.5 g/dL.

VSR repair was performed via median sternotomy under standard cardiopulmonary bypass. A 3- × 2-cm apical septal defect was closed using a double-patch technique reinforced with felt (Video 1). Bypass and crossclamp times were 97 and 64 minutes, respectively. No blood products were administered.

**Figure 3:**
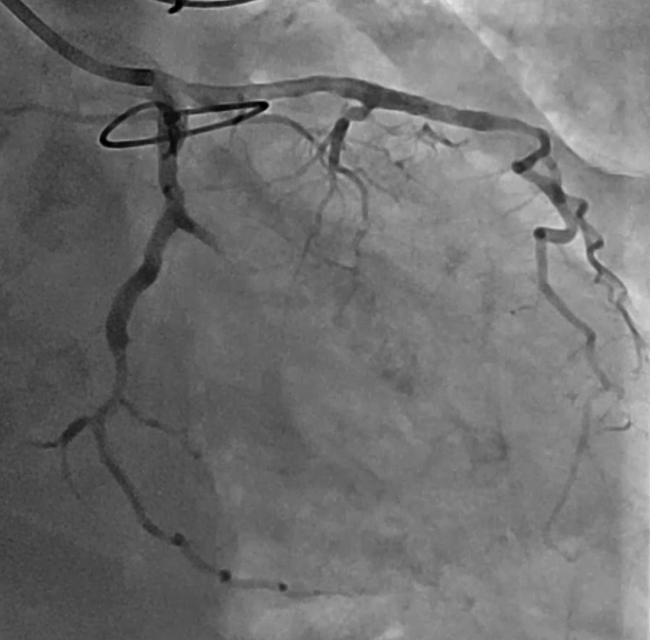
Coronary angiography after deferred percutaneous coronary intervention demonstrates Thrombolysis In Myocardial Infarction 3 flow with 0% residual stenosis in 7 and 14.

The patient was extubated 3 hours postoperatively, and IABP was removed on postoperative day (POD) 1 (hemoglobin 8.0 g/dL). Postoperative echocardiography demonstrated complete closure (Figure 2, *B*). Deferred PCI for 7 and 14 was performed on POD 15 when hemoglobin recovered to 11.9 g/dL and inflammatory markers had subsided (Figure 3). She was transferred to rehabilitation on POD 35 and remains asymptomatic nearly 3 years postoperatively. Institutional review board approval was waived. Written informed consent for publication was obtained.

## Discussion

Once VSR was identified, management shifted to a staged, blood-sparing plan tailored to transfusion refusal. Our JW program uses a structured, multidisciplinary protocol. For elective surgery, based on previous reports,^2^^,^^3^ we target preoperative hemoglobin ≥12 g/dL using intravenous iron, erythropoietin, vitamin B12, and folate, combined with hemodilutional autologous transfusion, cell-salvage reinfusion, and meticulous hemostasis. In this case, the same framework was applied pragmatically, proceeding to repair when hemoglobin and tissue integrity were acceptable.

The choice of mechanical support is pivotal. Although Impella (Abiomed) provides active unloading, studies show higher rates of bleeding, hemolysis, and transfusion than with IABP.^4^^,^^5^ For patients who JW, IABP offers balanced circulatory support while conserving hemoglobin. To reduce bleeding risk and procedural complexity under severe anemia, the initial operation was deliberately limited to septal repair. Meticulous hemostasis and restrained bypass and clamp times enabled smooth separation and early recovery, and deferred PCI after stabilization completed full revascularization.

Overall, this experience suggests a stepwise strategy—physiologic IABP bridging, blood-sparing surgery, and deferred PCI—may be a feasible option for selected post-MI VSR in JW. At our center, >60 transfusion-free cardiac operations in JW, including urgent cases, have yielded zero operative mortality, supporting the practicality of such an individualized approach.

## Conflict of Interest Statement

The authors reported no conflicts of interest.

The *Journal* policy requires editors and reviewers to disclose conflicts of interest and to decline handling or reviewing manuscripts for which they may have a conflict of interest. The editors and reviewers of this article have no conflicts of interest.

## Supplementary Data

Video 1 The entire surgical procedure is shown. Video available at: https://www.jtcvs.org/article/S2666-2507(25)00554-1/fulltext.
