# Supplementary figures and images for: Staged, blood-sparing management of postmyocardial infarction ventricular septal rupture in a Jehovah's Witness
Source: JTCVS Tech. 2025 Dec 3;35:102173. doi: 10.1016/j.xjtc.2025.102173 (PMC12881821; doi:10.1016/j.xjtc.2025.102173)

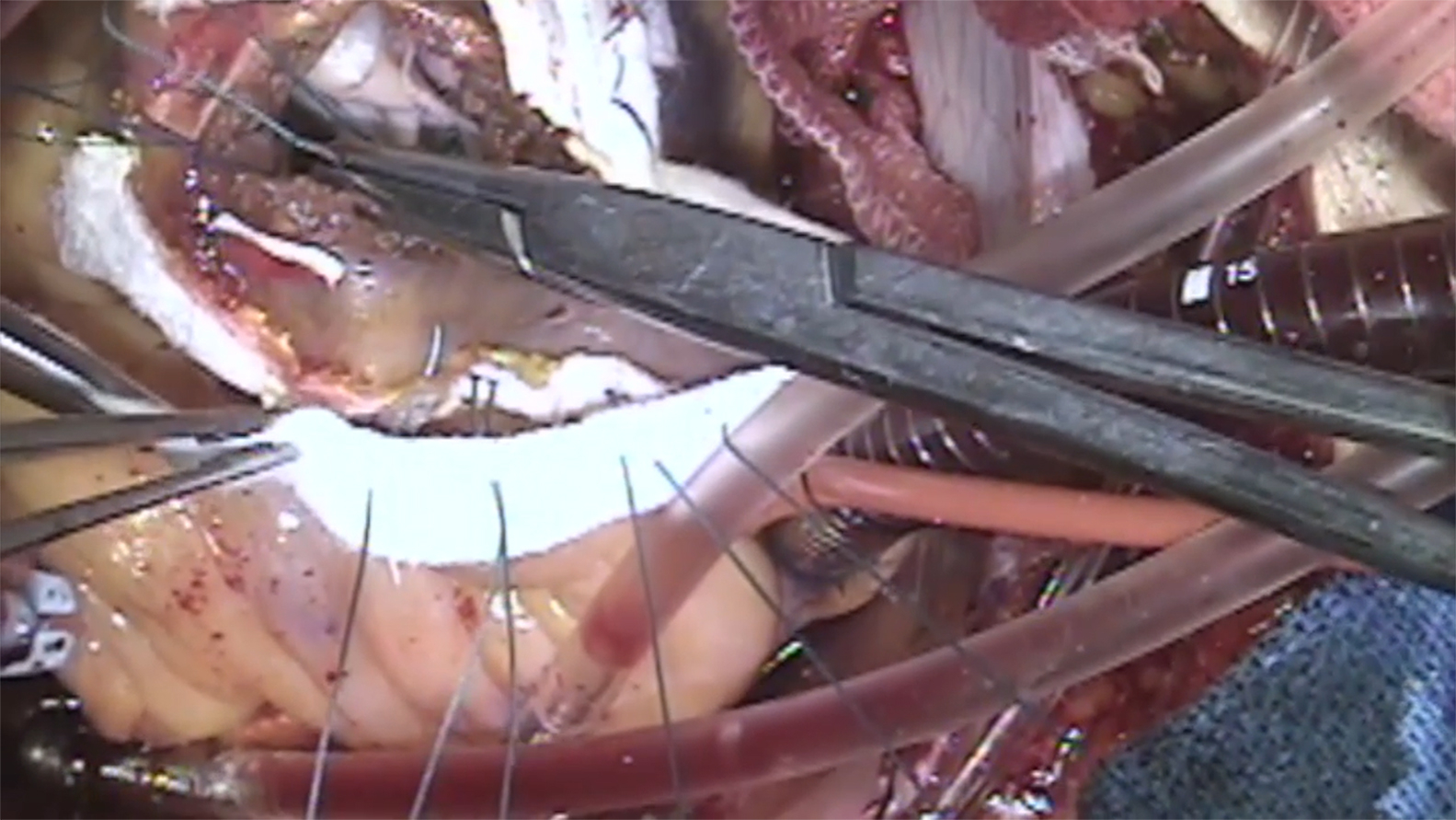

Supplement: Video 1 — The entire surgical procedure is shown. Video available at: https://www.jtcvs.org/article/S2666-2507(25)00554-1/fulltext. [file fx2.jpg]
